# Advances in single-cell RNA sequencing and its applications in cancer research

**DOI:** 10.18632/oncotarget.17893

**Published:** 2017-05-16

**Authors:** Sibo Zhu, Tao Qing, Yuanting Zheng, Li Jin, Leming Shi

**Affiliations:** ^1^ Center for Pharmacogenomics, School of Life Sciences and Shanghai Cancer Center, Fudan University, Shanghai, 200438, China; ^2^ Collaborative Innovation Center of Genetics and Development, Fudan University, Shanghai, 200438, China

**Keywords:** single cell, RNA sequencing, tumor, circulating tumor cell

## Abstract

Unlike population-level approaches, single-cell RNA sequencing enables transcriptomic analysis of an individual cell. Through the combination of high-throughput sequencing and bioinformatic tools, single-cell RNA-seq can detect more than 10,000 transcripts in one cell to distinguish cell subsets and dynamic cellular changes. After several years’ development, single-cell RNA-seq can now achieve massively parallel, full-length mRNA sequencing as well as *in situ* sequencing and even has potential for multi-omic detection. One appealing area of single-cell RNA-seq is cancer research, and it is regarded as a promising way to enhance prognosis and provide more precise target therapy by identifying druggable subclones. Indeed, progresses have been made regarding solid tumor analysis to reveal intratumoral heterogeneity, correlations between signaling pathways, stemness, drug resistance, and tumor architecture shaping the microenvironment. Furthermore, through investigation into circulating tumor cells, many genes have been shown to promote a propensity toward stemness and the epithelial-mesenchymal transition, to enhance anchoring and adhesion, and to be involved in mechanisms of anoikis resistance and drug resistance. This review focuses on advances and progresses of single-cell RNA-seq with regard to the following aspects:

1. Methodologies of single-cell RNA-seq

2. Single-cell isolation techniques

3. Single-cell RNA-seq in solid tumor research

4. Single-cell RNA-seq in circulating tumor cell research

5. Perspectives

## INTRODUCTION

RNA sequencing (RNA-seq) has recently been developed as a powerful tool for investigating the intracellular transcriptome based on next-generation sequencing (NGS) [1]. “Salt-and-pepper” variation is ubiquitous throughout a cell population or tissue, and this results in cell-to-cell transcriptomic diversity [2]. However, most current transcriptomic studies are performed on a bulk level and typically investigate the average of variable transcriptomes from millions of cells. Moreover, it is difficult to evaluate dynamic changes in an individual cell (10–20 pg total RNA) [3, 4] with the current population-based RNA-seq methods, which for mammals, require hundreds of thousands to millions of cells (0.1–5 μg total RNA) [5, 6]. In contrast, single-cell RNA-seq (scRNA-seq) has the potential to easily overcome these obstacles.

Since 2009, a plethora of scRNA-seq technologies have been developed, providing an unbiased measurement of expression profiles at a single-cell resolution. In an effort to enhance RNA detection sensitivity and full-length transcript quantitation and to reduce technical variability, massively parallel sequencing, *in situ* sequencing and multi-omic sequencing are enabling in-depth identification of new cell types, sub-populations and biomarkers. In terms of single-cell manipulation and isolation from a potentially heterogeneous population of different types of cells, approaches such as micromanipulation, microfluidics, fluorescence-activated cell sorting (FACS), and laser-capture microdissection (LCM) are well developed and applied. In addition, computational tools have emerged in a short period of time to assess the functional implications of stochastic transcription by dissecting variabilities and background noises such as those due to expression changes of genes involved in cell cycle [4, 7, 8].

The diverse applications of scRNA-seq include embryogenesis and stem cell differentiation, organ development, immunity, whole-tissue subtyping, neurobiology and tumor biology. Notably, cancer research is becoming even more intriguing, as intratumoral heterogeneity and the tumor microenvironment can now be studied with scRNA-seq. Solid tumors, cell lines, and circulating tumor cells (CTCs) are hot topics in the single-tumor cell research arena, showing a powerful capacity to reveal transcriptomic heterogeneity, signaling pathways related to drug resistance, immune tolerance and intratumoral heterogeneity. In this review, we mainly discuss the significant progresses in the scRNA-seq and its applications in cancer research.

### Advances in single-cell RNA sequencing technologies

Single-cell RNA-seq was first reported in 2009 by Tang et al. for analyzing the mouse blastomere transcriptome at a single-cell resolution [5] and many protocols with pros and cons have been developed (Table 1). Islam et al. then developed the single-cell tagged reverse transcription sequencing (STRT-Seq) method by adopting a template switching oligonucleotide (TSO) to barcode the 5′ end of transcripts, allowing for unbiased amplification in comparisons across multiple samples [9]. Ramsköld et al. applied both a TSO in the Smart-Seq protocol to obtain full-length cDNA as well as the transposase Tn5 to barcode 96 samples. This method successfully evaluated distinct biomarkers, isoforms and single nucleotide polymorphisms (SNPs) for sequencing of CTC RNA from melanoma patients [10]. Later, Picelli et al. introduced Smart-Seq2, a modified protocol for Smart-Seq, resulting in higher sensitivity and improved coverage and accuracy using the locked nucleic acid (LNA), a modified inaccessible RNA nucleotide [11]. Tamar et al. established a Cel-Seq protocol via an *in vitro* transcription (IVT) technique that linearly amplified mRNA from single cells in a multiplexed barcoding manner [2, 12]. Pan et al. adopted rolling circle amplification (RCA) in single-cell analysis, a whole transcriptome amplification method for small amounts of DNA, and Lee et al. applied this method to FISSEQ *in situ* single-cell RNA seq [13, 14]. Moreover, Islam et al. tagged cDNA with unique molecule identifiers (UMI), providing a powerful tool for adjusting amplification bias, enhancing sensitivity and reducing background noise [3]. Achieving 96 single-cell parallel Smart-Seq2-based RNA-seq, Pollen et al. devised the microfluidic system Fluidigm C1 [15]. Two similar droplet-based massively parallel single-cell RNA-seq techniques, namely, Drop-Seq and Indrop-Seq by Klein et al. and Macosko et al., respectively, were released in May, 2015 [16, 17]. These techniques allowed several thousands of cells to be sequenced in a unique barcode-wrapped droplet. Fan et al. further established a massively parallel single-cell RNA-seq protocol facilitated by magnetic beads and combining cell capture and poly(A) selection, which could analyze up to 100,000 cells in microwells [18]. Fan et al. also achieved single-cell circRNA sequencing using a single-cell universal poly(A)-independent RNA sequencing (SUPeR-Seq) protocol [19].

**Table 1:** Main contributions to scRNA-seq technologies

Year	First Author	Protocol	Significance
2009	Tang [5]	scRNA-seq	First single cell RNA sequencing method
2011	Islam [9]	STRT-Seq	5′ sequencing with Template Swithing Oligo
2012	Ramsköld [10]	Smart-Seq	Full length mRNA sequencing
2012	Hashimshony [12]	Cel-Seq	*In vitro* transcription, linear amplification
2013	Picelli [11]	Smart-Seq2	Enhanced single cell RNA-seq sensitivity
2013	Pan [13]	RCA	Total RNA sequencing with Rolling Circle Amplification
2014	Lee [14]	FISSEQ	*In situ* single cell RNA-seq
2014	Islam [3]	UMI	Higher sensitivity by Unique Molecule Identifier
2014	Pollen [15]	Microfluidics	Massively paralleled, 96 cells per batch
2015	Klein [16]	inDrop-Seq	Massively paralleled, 3000 cells per batch
2015	Macosko [17]	Drop-Seq	Massively paralleled, 44800 cells per batch
2015	Fan [18]	Cyto-Seq	Massively paralleled, 10000–100000 cells per batch
2015	Fan [19]	SUPeR-Seq	circRNA sequencing
2015	Macaulay [22]	G&T-Seq	Simultaneous sequencing on genome and transcriptome
2016	Thomsen [20]	FRISCR-Seq	scRNA-seq after staining and FACS
2016	Hu [21]	scMT-Seq	Simultaneous sequencing on transcriptome and methylome
2016	Hou [23]	scTrio-Seq	Simultaneous sequencing on CNV, transcriptome and methylome
2016	Habib [24]	Div-Seq	*In situ* single nucleus RNA sequencing
2016	Nichterwitz [33]	LCM-Seq	*In situ* RNA-seq with laser capture microdissection
2016	Faridani [34]	Small RNA-seq	Analysis of microRNAs, tRNAs and small nucleolar RNAs

To profile primary human radial glia, intracellular staining combined with fixed and recovered intact single-cell RNA-seq (FRISCR-Seq) was developed by Thomsen et al., with little bias and similar gene expression yield, even when fixation and purification were introduced [20]. Macaulay et al. were the first to simultaneously conducted a single cell's genome and transcriptome sequencing by G&T-Seq, and Hu et al. simultaneously sequenced the methylome and transcriptome of a single cell using the single-cell methylome and transcriptome sequencing (scMT-Seq) technique [21, 22]. Hou et al. invented single-cell triple-omics sequencing (scTrio-seq), which simultaneously analyzed genomic copy-number variations (CNVs) and the DNA methylome and transcriptome from individual single hepatocellular cancer cells [23]. More recently, Habib et al. developed Div-Seq, a scalable single-nucleus RNA-seq (sNuc-Seq)-based technique to identify closely related hippocampal cell types and track dynamic changes in the newborn neuron transcriptome with high sensitivity [24].

In terms of bioinformatics, tools for analyzing RNA-seq data from bulk populations can partly be applied to transcriptomic data at the single-cell level, yet many new computational strategies are needed to normalize raw data and exploit featured transcriptional kinetics [7]. Buettner et al. revealed that confounders such as the cell cycle, technical noises and biological variabilities contribute to cell-to-cell variation. However, cell cycle noises can be reduced by the single-cell latent variable model (scLVM), a cell cycle-based expression profile correction method [7]. Other efforts have also been made to reduce single-cell transcriptomic noises, including pathway and gene set overdispersion analysis (PAGODA) by FAN et al., inferring stochastic transcriptomic kinetics by Kim et al. and single-cell differential expression (SCDE) by Kharchenko et al., which addresses data correction using previously-annotated pathways and gene sets, as well as automatically-detected gene sets amplification bias and dropout events via a Bayesian approach [25–27]. Stubbington et al. developed the TraCeR method, which was able to reconstruct full-length, paired T cell receptor sequences from V(D)J regions to link single T lymphocyte specificity with functional responses as well as the transcriptional landscape [28].

Mitochondrial RNAs may cause biased interpretation of sequencing data, and have been observed to increase in broken cells or cells undergoing apoptosis, due to the loss of cytoplasmic RNA. However, reports showed that models like support vector machine (SVM) can filter out confounding samples and reduce artifacts effectively [3, 29]. Finally, remodeling of single-cell subpopulations, trajectories and bifurcation events can be achieved by many microevolution analyses. Without given temporal information, Moncole and Waterfall adopted independent component analysis (ICA) and k-means to produce clusters and infer a pseudo temporal ordering by minimum spanning tree (MST) [30, 31]. Given time course data, single-cell clustering using bifurcation analysis (SCUBA) is able to detect bifurcation events based on stochastic differential assumption [32].

### To summarize, several landmarks achieved using the above technical advances are described below

#### Poly(A) selection-based reverse transcription

A poly(A)-based method initiated the first single-cell RNA-seq technique, and this method is now widely applied as a standard protocol in single-cell RNA-seq. To synthesize first-strand cDNA in a microliter or nanoliter reaction, oligo-dT primers are applied for hybridization of poly(A)-tailed mRNA from the 3′ end. In this step, most undesirable tRNAs and rRNAs are removed, though many non-(A)-tailed lncRNAs are also excluded. Followed by incorporation with proper adapters, cDNA is amplified by several orders of magnitude using PCR-based amplification, IVT or RCA. Typical examples include Tang et al.'s scRNA-seq and Quartz-Seq [33].

#### Full-length transcript sequencing

This technique has a significant impact on research into dynamic changes in exons, introns, and alternative splicing, as these events are observed at a single-cell resolution. For example, in the Smart-Seq2 protocol, a typical full-length single-cell RNA-seq technique, cDNA is not only synthesized from the 3′ end, but 5′-3′ coverage is also guaranteed by TSO, which eliminates 3′ bias. Apart from TSO-based techniques, Phi29-mRNA amplification (PMA) and semi-random primed PCR-based mRNA transcriptome amplification (SMA) amplify cDNA by rolling amplification using the Phi29 enzyme and semi-random primers, respectively [34]. Representative methods include PMA/SMA-Seq, Multiple Annealing and Looping-Based Amplification Cycles (MALBAC)-based RNA-seq [35], Smart-Seq and Smart-Seq2.

#### Adoption of a unique molecular identifier

Another milestone in single-cell RNA-seq is the application of UMI for identifying the exact number of transcripts in a cell and thus quantifying sensitivity. As first reported by Islam et al., UMI comprises a 5 base-pair-length random nucleotide, which is incorporated into the 5′ end of cDNA during reverse transcription. Therefore, the absolute scale of measurement is achieved by counting the number of known spike-ins. Noise introduced by reverse transcription can also be eliminated by UMI incorporation. Typical examples include Islam's single-cell RNA-seq, Drop-Seq, Indrop-Seq and Cyto-Seq.

#### Massively parallel single-cell RNA-seq

This highly multiplexed profiling technique shows the potential to not only reduce technical noise during library preparation but to enhance reproducibility. Currently high-throughput single-cell RNA-seq approaches are achieved using microfluidics and robotics, collecting hundreds to thousands of cells per batch at a much larger scale and in a faster process than manual selection. The first reported massively parallel single-cell RNA-seq, namely MARS-Seq, sorted and classified cell types ab initio from more than 4,000 cells from splenic tissues using an unsupervised clustering algorithm [36]. Fluidigm C1 facilitated single-cell cDNA library preparation with reaction lines in which 96 individual samples were reverse transcribed. As the total volume of a library preparation chamber is approximately 300 nanoliters, less reagent is consumed compared to a regular setup (25 microliters). Droplet-based approaches such as Drop-Seq and inDrop-Seq as well as 10× Genomics can prepare thousands of libraries in even smaller droplets [37]. However, there are also disadvantages to throughput enhancement: as the sequencing depth and coverage are much lower, quantitation normally relies on 3′ reads. MARS-Seq, Fluidigm, Drop-Seq, inDrop-Seq and 10× Genomics are included in this category.

#### Multi-omic simultaneous sequencing

These multiple layer profiling methods are capable of simultaneously obtaining information regarding the transcriptome, methylome and genome. By measuring more than 6,000 promoter methylation sites and 4,600 transcripts in an individual cell, scMT-Seq, a combination of reduced representation bisulfite sequencing (RRBS) and Smart-Seq2, revealed the regulative relationship between the epigenomic status and expression pattern. Using scTrio-Seq, Hou et al. not only showed positive correlations between CNVs and expression (Pearson r = 0.73) but also demonstrated a powerful tool for simultaneously obtaining information from three cellular omes. Intriguingly, this study also validated that promoter methylation negatively correlates with gene expression, whereas methylation on the gene body promotes it. Correlations among -omes provide new insights into the dynamics of gene regulation. More recently, Faridani et al. described a novel technique for simultaneous detection of microRNAs, fragments of tRNAs and small nucleolar RNAs from single cells at a low-input level, shedding new light on non-coding RNA [38]. CircRNA-seq, small RNA-seq, scMT-Seq, scTrio-Seq and G&T-Seq have all been reported in this research field.

#### Tissue decomposition single-cell RNA-seq

This approach is now widely adopted to define new cell and tissue types through unsorted single-cell RNA-seq and unsupervised digital transcriptome clustering. Since the development of bulk RNA-seq, human, mouse and rat body map projects and transcriptomic landscapes have been plotted at the organ and tissue levels [39–41]. However, combined with tissue decomposition, single-cell sorting, microfluidic approaches and single-cell RNA-seq are now able to distinguish rare cell subtypes in solid organs. For instance, Jaitin et al. revealed heterogeneity in dendritic cell subpopulations and new cell types in an LPS stimulation experiment, and Treutlein et al. discovered a new cell type on day E18.5 during mouse distal lung epithelia development. Zeisel et al. and Tasic et al. molecularly classified the mouse hippocampus and cortex through single-cell RNA-seq, and a diversity of neuron and glia subtypes were identified. La Manno et al. studied ventral midbrain development, identifying 25 and 26 clusters in mouse and human, respectively [36, 42–45]. These findings undoubtedly advance the fields of development biology, histopathology, immunology, neurology and cancer biology.

#### *In situ* single-cell RNA-seq

This revolutionary technique combines analysis of the single-cell transcriptome and its spatial distribution. In the process of *in situ* scRNA-seq, cells of interest are identified by specific staining such as fluorescence, and then followed by manual or robotic picking. Fluorescent *in situ* RNA sequencing (FISSEQ) was first reported to have potential for *in situ* investigations of cellular phenotypes, gene regulation, and the cellular microenvironment [14]. Achim et al. successfully sequenced tissues of interest from P. dumerilii using whole-mount *in situ* hybridization (WMISH) and parallel single-cell RNA-seq (Fluidigm C1) [46]. Similar approaches include Nichterwitz's LCM-Seq of frozen mouse spinal sections [47] and Lovatt's transcriptome *in vivo* analysis (TIVA) of live tissues [48]. Notably, electrophysiological phenotype recording-oriented single-cell RNA-seq (Patch-Seq) is achieved via integration of patch-clamp and cell aspiration techniques [49].

Generally, 0.05~0.1 million mapped reads are sufficient to distinguish cell types as reported using Fluidigm C1, or Drop-seq or 10× Genomic protocols [17, 50, 51]. However, deeper sequencing is also seen in the ab initio cell identification, mutation calling and identification of RNA splicing events, with reads ranging from 5~20 million per sample [10, 42, 52, 53]. By far, there is no research showing difference of quantitation results between single-end and paired-end reads. However, paired-end mode shows advantage at fusion or splicing discoveries but generates few reads. Multiplexing of samples per lane depends on the desired sequencing depth and lane capacity of each instrument. For example, normally 0.5~2 million reads per sample can be obtained from a pooled library with 96~384 barcodes sequenced by a HiSeq 2500 sequencer.

### Single-cell isolation techniques

Because single-cell RNA-seq is based on an individual cell or subcellular apparatus, e.g., the nucleus, isolation of target cells from complex and heterogeneous tissues is an initial and essential step. Manual picking, single-cell FACS, microfluidics and LCM are currently adopted as mainstream single-cell isolation methods. Here we briefly introduce the principles of these techniques and discuss their advantages or disadvantages (Table 2).

**Table 2:** Advantages and disadvantages of single cell isolation methods

Isolation Methods	Advantages	Disadvantages
Manual Picking	Low cost, accurate isolation	Low throughput, low sensitivity
Single cell FACS	Surface marker sorting	Low capture rate on rare cells
Microfluidics	High sensitivity, High throughput, Automatic library preparation	Marker based sorting is not applicableAffected by cell size (Fluidigm)Doublet (Drop-Seq)False Pos/Neg (CTC-Chips)
LCM	Cell dissected from spatial origin	Low accuracy, currently only available to frozen sections

CTC isolation, a specialized application in this field, is achieved by employing surface marker detection, size screening, gradient separation and cluster capture. On one hand, CTCs have significant diagnostic value because these cells are rare in a patient's blood; on the other hand, methods with higher sensitivity and specificity need to be developed. Widely adopted CTC isolation methods include magnetic bead capture, microfluidic enrichment, size filtering and image-based selection.

### Manual picking

As a user-friendly method, manual picking of single cells has been employed in many protocols, such as in Smart-Seq, Smart-Seq2 and Cel-Seq library preparation. Normally, a mouth pipette or micropipette is used to select a target single cell under a microscope and place it into lysis and reverse transcription tubes [10–12, 53]. In addition, rare cells such as CTCs can be fluorescently stained for surface markers, and doublets are avoidable through manual inspection. However, compared to automated cell-picking devices, this method has neither a satisfying sensitivity nor a high-throughput and speed capacity per batch [3, 54, 55].

### Microfluidics

By analyzing physiological properties such as the size, charge, magnetism, and surface markers of different cell types, microfluidic devices enable efficient single-cell separation, cell culture, and library preparation in integrated fluidic microsystems [15, 56, 57]. DEP-Array, Fluidigm, CTC-iChip, Cyto-Seq and Drop-Seq belong to this category [58]. In terms of CTC application, antibody-specific beads or image-based microfluidic devices, including CTC-iChip, HbCTC-Chips, MagSweeper and CellSearch systems, are adopted to isolate these specific rare cells. Advantages of microfluidics include not only simultaneous single-cell library preparation in a higher throughput manner than manual manipulation but also higher sensitivity and reproducibility [54]. Dramatic improvements are needed for multi-marker-based cell sorting to reduce false positive/negative and doublets in downstream sequencing [16, 59].

### Fluorescence-activated cell sorting (FACS)

This multi-channel, fluorescent antibody dye-based cell sorting approach has been broadly applied in many single-cell transcriptome studies [24, 60–62]. Using uniquely tagged fluorophores, cell subpopulations of interest are sorted within only minutes into a 96-well or 384-well plate for library preparation [60, 63, 64]. However, on the one hand, rare CTCs, which have a frequency below one in a million, are not easily detected and isolated by current FACS methods; on the other hand, flow cytometry is not able to handle a starting cell-suspension volume less than several microliters [64, 65].

### Laser-capture microdissection (LCM)

Under direct microscopic visualization, LCM can harvest cells of interest or isolate specific cells by cutting unwanted tissues in either formalin-fixed paraffin-embedded (FFPE) or cryostat sections using UV or infrared (IR)-coupled microscopy. Commonly used LCMs include LDM systems by Leica, PixCell systems by Molecular Devices, and photoactivated localization microscopy (PALM) systems by Zeiss [66, 67]. Guided by a target beam, a minimal 7.5-μm spot-sized laser is rapidly pulse-fired at frozen sections, FFPE sections, direct smear or Touch Preps [68]. Research shows rather low RNA integrity numbers (RINs), 2.1–2.4 for FFPE samples [69], though RNA remains intact, with an RIN > 8.5, on frozen slides [70, 71]. The first application of LCM-based single-cell RNA-seq was performed and named LCM-Seq, showing a high gene detection rate, reproducibility and advantages in mouse and human neuron *in situ* heterogeneity analyses [47]. As a prospective visualized single-cell isolation method, improvements regarding precision and noise reduction during UV/IR dissection are needed for the LCM technique [72].

### CTC detection and isolation

CTCs are extremely rare, with only one tumor cell per billion normal blood cells in the circulation, and exist in a single or cluster form [73, 74]. Several methods have been developed to identify CTCs according to different principles, such as cell marker-based detection (EpCAM, CK or other cancer-specific antigens), size filtration, gradient separation, and luminescence reporter systems. Membrane marker detection and isolation approaches are currently in wide use. Normally 1~10 mL of freshly taken whole blood is loaded into an anticoagulant tube. Then CTCs are either directly identified by positive selection, or enriched by negative selection, followed by marker-based identification. This technique category includes the FDA-approved CellSearch system by Johnson & Johnson, the dielectric field array-based automatic cell sorting system DEP-Array by Silicon Biosciences, the continuously updated CTC-Chips by Harvard University, and image recognition and manipulation-facilitating CellCelector by Lab Solutions [10, 75–80]. In addition, physical separation methods such as Cluster-Chip by Harvard University, isolation by size of epithelial tumour cells (ISET) by Rarecells, CellSieve by Creatv MicroTech, and the OncoQuick gradient reagent by Greiner Bio-one have been adopted for diagnostic and academic fields [79, 81–84]. (Table 3).

**Table 3:** Methods for the identification and isolation of circulating tumor cells (CTCs)

CTC Identifier	Company or Organization	Isolation mechanics	Blood (mL)	Principle
CellSearch [80]	Johnson & Johnson	Antibody conjugated beads	7.5	Membrane antigen detection
LiquidBiopsy [85]	Cynvenio Biosystems Inc	Antibody conjugated beads	7.5	
MagSweeper [86]	Illumina	Antibody conjugated beads and magnetic rods	7.5	
CTC-Chips [87–89]	Harvard Medical School	Antibody conjugated beads and coated rods	1.0–3.0	
ICeap [90]	Tohoku University	Antibody conjugated beads and FACS	4.0	
IsoFlux [91]	Isoflux	Antibody conjugated beads and microfluidics	7.5	
FACS [92, 93]	BD/Beckman Coulter	Fluorescence activated single cell sorting	7.5	
DEPArray [76]	Silicon Biosciences	Image based dielectrophoresis microfluidics	10^4^ cells*	
CellCelector [77]	Automated Lab Solutions	Image based automatic single cell manipulation	10^3^~10^4^ cells*	
Accu-Cyte [94]	Rarecyte	Image based automatic single cell manipulation	7.5	
SET-iFISH [95]	Cytelligen	Image based manual single cell manipulation	6.0	
Cluster-Chip [83]	Harvard Medical School	CTC cluster trap	4.0	Physical separation
ISET [81]	Rarecells	Size filter	10.0	
CellSieve [82]	Creatv MicroTech	Size filter	7.5~10.0	
OncoQuick [96]	Greiner Bio-One	Gradient separation	15.0–30.0	
Spiral biochip [97]	UNSW/MIT/NUS	DFF based spiral microfluidics	7.5	
Microchannel Chip [98]	Ventana Medical Systems	Size filtration based microfluidics	2.0	
Vortex Chip [99]	UCLA	Wall shear stress microfluidics	7.5	
CTC-RV [100]	Johns Hopkins University	Tissue specific adenovirus reporter system	1.0	Fluorescence reporter
Ad5/35E1aPSESE4 [100]	NCC, South Korea	Tissue specific adenovirus reporter system	5.0	

*CTC enriched in advance.

### Single-cell RNA-seq for deciphering the solid tumor architecture

**Figure 1:**
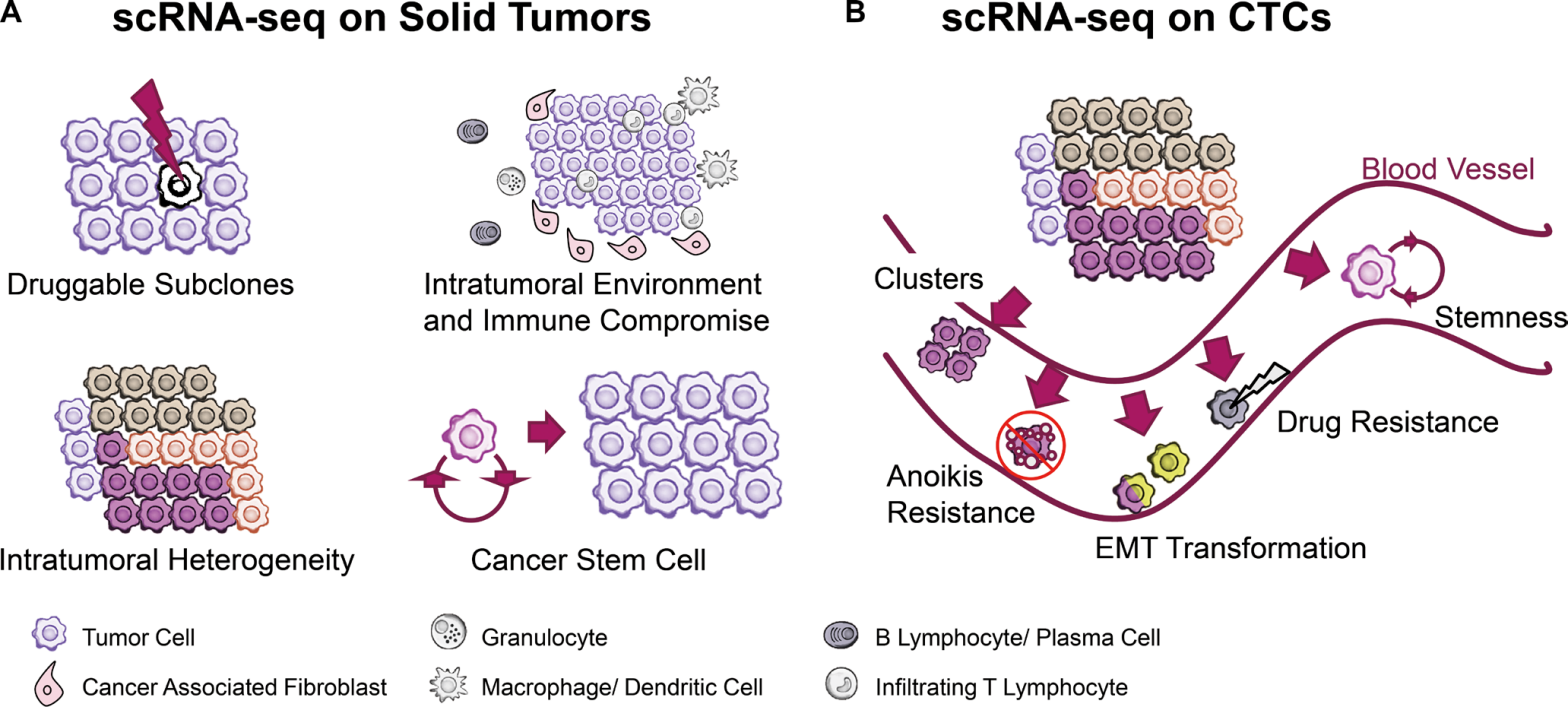
scRNA-seq technology facilitates cancer research when coping with solid tumor tissues and circulating tumor cells (**A**) Findings of abnormal cell-to-cell interaction, drug resistance, and intratumoral immune microenvironment are achieved with tissue decomposition technologies. (**B**) Circulating Tumor Cells (CTCs) were captured and sequenced to explain the rationale underlying anoikis resistance, cluster induced metastasis, EMT transformation and stemness.

Cancer heterogeneity generating from diverse single-tumor cell subpopulations imposes great challenges for clinical diagnosis and treatment. Single-cell RNA-seq has the potential to identify genomic and transcriptomic information from intratumoral cells and to provide new insight into tumor heterogeneity. Recently, single-cell transcriptomic analysis has enabled the functional characterization of abnormal cell-to-cell interactions, drug resistance, the intratumoral architecture and the immune microenvironment with solid tissue decomposition and isolation methods. (Figure 1A)

### Glioma

Patel et al. resected and dissociated five human glioblastomas and generated single-cell RNA-seq data using the SMART-seq protocol. Unanticipated heterogeneity was revealed in 430 single cells showing transcriptional diversity related to oncogenic signaling, proliferation, complement system and immune response, and hypoxia. Therapeutic targets such as EGFR, PDGFRA, PDGFA and other proteins pertinent to glioma were also expressed mosaically. It is also worth noting that evidence of stemness *in vivo* was found to correlate with transcription factors (TFs), such as POU3F2, NFIA, and NFIB [61]. Tirosh et al. profiled 4,347 single cells from six IDH1 or IDH2 mutant human oligodendrogliomas and revealed a rare undifferentiated subtype showing stem cell potential alongside astrocyte and oligodendrocyte subpopulations. Cell-cycle gene expression signatures distinguishing G1/S and G2/M were applied to show proliferative and self-renewal potential in 10% of the population. This finding supported the cancer stem cell hypothesis at the single-cell level, with essential implications in glioma therapy [85].

### Melanoma

Tirosh et al. analyzed 4645 single cells from 19 melanoma patients by FACS and Smart-Seq2 RNA-seq. Several cell subpopulations were identified, including tumor cells, stromal cells, cancer-associated fibroblasts (CAFs), endothelia, T cells, B cells, and macrophages. Intrapatient and interpatient heterogeneity revealed the MITF-AXL drug resistance axis, whereas KDM5B was found to be inversely related to the cell cycle and tumor proliferation, i.e., showing a static status. Analysis of infiltrating immune cells further showed that enhanced cytotoxicity and proliferation of CD8+T cells were accompanied by several elevated exhaustion markers, such as PD-1, TIM-3 and CTLA-4. This study not only demonstrated heterogeneity between tumor cells but characterize the intratumoral ecosystem with consequences for future targeted and immune therapy [60].

### Hepatocellular carcinoma

Hou et al. simultaneously analyzed genomic CNVs and the epigenome and transcriptome from individual single hepatocellular cancer cells using scTrio-Seq [86]. Two populations of single cancer cells were distinguished through a combination of CNV, methylation and expression profiling. Differentially expressed genes from one subpopulation were enriched in the acute inflammatory response, innate immune response, and complement activation pathways, showing immune responsiveness; another population expressed a greater degree of invasive-cell markers and was thus more likely to evade immune surveillance.

### Lung cancer

Kim et al. analyzed the transcriptome of single cells from patient-derived xenograft (PDX) tumor tissue with lung adenocarcinoma origin. Intra-tumoral SNV and expression heterogeneity were revealed using a collection of highly heterogeneous genes, including KRASG12D, across cells. The study also reported that after *in vitro* chemotherapeutic screening, the drug-resistant population exhibited downregulation of cell cycle-related genes, whereas ion transporter activity was enhanced [87]. Suzuki et al. sequenced 336 LUAD cells from 7 different cell lines using the Fluidigm C1 technique to reveal transcriptomic changes before and after multi-tyrosine kinase inhibitor vandetanib treatment [88]. Target genes of vandetanib such as EGFR and RET were slightly affected by treatment with the drug, though relative expression of housekeeping genes and ribosomal genes was significantly reduced. The authors inferred that a robust transcriptional control of these target genes may not be allowed to alter their expression during acquisition of drug resistance. Interestingly, based on the PCA plot, the vandetanib-sensitive cell line remained together with the same cell line after drug treatment, whereas the drug-resistant population was separated.

### Renal carcinoma

Kim et al. performed single-cell RNA-seq on metastatic renal cell carcinoma, primary tumor PDX, and metastatic PDX samples from a renal carcinoma patient. Principal component analysis revealed that cells with metastatic foci were distant from the primary tumor, whereas PDX samples were close to metastatic foci, indicating the reliability of the PDX model in metastasis research. In addition, single-cell sequencing revealed that the two originally mutually exclusive signaling pathways, i.e., EGFR-activated and Src/FAK-activated subsets of cells, were masked in the population. Through experimental validation, a combination drug administration experiment exhibited a greater inhibitory effect on tumor growth and induced more apoptosis compared to monotherapy both *in vitro* and *in vivo* [52].

### Single-cell RNA-seq for understanding the nature of CTCs

CTC detection as a promising liquid biopsy has been employed in many cancers for diagnosis and prognosis purposes. More than 19,000 publications can be retrieved in PubMed using the key phrase “circulating tumor cell” (Feb, 2017). Early CTC studies focused on cell number in metastatic breast cancer patients using CellSearch systems to stain and count cells. A predictive model was established by associations between survival rates and CTC numbers [89]. Similar results that a higher number of CTCs indicates a poorer prognosis have been validated in many other cancer types, such as prostate, lung, colorectal and ovarian cancers [90].

With the development of scRNA-seq, it is now possible to observe changes in the transcriptome, alternative splicing and single-nucleotide variations in an individual CTC and to perform detailed studies on the mechanisms of anoikis resistance, metastasis, drug resistance, cancer stemness and other common traits in cancer (Figure 1B). Below, we discuss some of the progresses made in patient-oriented CTC research (Table 4).

**Table 4:** Transcriptomic studies of CTCs

First Author	Year	CTC Isolation Marker/Device	Library / Sequencer	Cancer Type	Significance
Yu [107]	2012	CK/EpCAM/HbCTC-Chip	SuperscriptIII+TdT/Helicos	Pancreatic Carcinoma	WNT pathway in anoikis and metastasis
Ramsköld [10]	2012	EpCAM/MagSweeper	Smart-Seq/Hiseq2000	Melanoma	First CTC single cell RNA-seq
Yu [108]	2013	CK/HER2/HbCTC-Chip	SuperscriptIII+TdT/Helicos	Breast Cancer	EMT evidence in CTCs
Ting [73]	2014	CK/CTC-iChip	Tang's scRNA-seq/SOLiD	Pancreatic Carcinoma	SPARC gene promotes metastasis, CTC subtyping
Aceto [78]	2014	EpCAM/HER2/CTC-iChip	SuperscriptIII+TdT /SOLiD	Breast Cancer	Higher metastasis in cluster than single cell
Sarioglu [83]	2015	Cluster-Chip	SuperscriptIII+TdT /SOLiD	Multiple Cancer Types	Macrophage like cells found in CTC clusters
Hwang [109]	2015	Fluorescence Microscopy	SENSE/Hiseq2000	Prostate Cancer	PSA promoter applied in tracking and CTC Staining
Miyamoto [110]	2015	EpCAM/CDH11/CTC-iChip	Tang's scRNA-seq/SOLiD	Prostate Cancer	WNT pathway mediated drug resistance
Grillet [111]	2016	RosetteSep	N/A	Colon Cancer	Stemness in colorectal carcinoma CTCs
Jordan [112]	2016	EpCAM/HER2/CTC-iChip	Tang's scRNA-seq/Hiseq2000	Breast Cancer	Drug resistance and HER2 expression

### Pancreatic ductal adenocarcinoma

In an early study, Yu et al. collected and sequenced CTCs from pancreatic ductal adenocarcinoma (PDAC) using a combination of CD45- and EpCAM+ fluorescence-aided manual picking and HbCTC-Chip enrichment techniques. Activation of the non-classical WNT pathway was found to play an important role in anoikis resistance, enhanced anchorage-independent sphere formation, and metastasis potential, which was thought to be associated with fibronectin up-regulation [80, 91]. Using the scRNA-seq technique, CTCs from 75 pancreatic cancer KPC mice were sequenced and classified on the basis of single-cell stratification; CTC-c, CTC-plt and CTC-pro types were subclassified according to surface markers. The findings suggested the heterogeneity of the CTC transcriptome and revealed that the extracellular matrix gene SPARC promotes distant metastasis [92].

### Melanoma

Cann et al. utilized human LNCaP, PC-3 and T24 cell lines to mimic human CTC incorporation into the peripheral blood of healthy volunteers and applied MagSweeper and CellSearch techniques to isolate simulated CTCs from the blood samples and from prostate cancer patients. Smart-Seq single-cell RNA-seq technology was also used and the reliability of the MagSweeper technology verified; the integrity of the CTC transcriptome RNA was greater than that of the simulated cell lines, suggesting that CTCs have a short half-life [93]. Ramsköld et al. employed the MagSweeper apparatus to sort melanoma CTCs and compare differentially expressed genes between CTC and primary melanoma cell lines using Smart-Seq single-cell sequencing, finding 9 novel upregulated membrane surface antigen candidates [10].

### Breast cancer

Using the HbCTC-Chip enrichment technique, Aceto et al. found that clusters of CTCs were more likely to generate lung metastases than single free CTCs in a breast cancer xenograft mouse model. Using single-cell RNA-seq, it was found that the CTC clusters exhibited higher expression of desmosomal proteins and adhesion-connexin genes, such as desmoplakin, than single CTCs. Interestingly, when Plakoglobin expression was downregulated by RNA interference, fewer CTC clusters were found in the model, and the metastatic rate was greatly reduced [78]. Moreover, Sarioglu et al. designed the Cluster-Chip system to specifically capture CTC clusters from cancer patients and found that in addition to CTC-specific biomarkers, macrophage/monocyte markers were also expressed by the CTC clusters, showing that tissue-derived macrophages migrated with these CTC clusters [94]. Yu et al. employed HbCTC-Chip to capture CTCs from 11 patients with breast cancer; during the course of follow-up, RNA-FISH revealed that the epithelial-mesenchymal transition (EMT) state of CTCs from patients with advanced disease were of the M-type (mesenchymal), whereas the remission stage was associated with E-type (epithelial) cells. CTC single-cell RNA-seq from a 5-point follow-up patient showed that E- and M-type transcripts, altered by the TGF-b pathway and the FOXC1 transcription factor, could be transformed at different stages of the treatment course [95].

### Prostate cancer

Miyamoto et al. isolated 77 CTCs from 13 prostate cancer patients and performed single-cell RNA-seq, and the results showed CTC heterogeneity rich in many aspects, such as androgen receptor mutation, androgen receptor splicing, and ncWNT pathway activation. In this study, some patients demonstrated resistance to castration treatment, and their CTCs were activated for the non-classical WNT pathway. The results showed that the WNT5A gene was highly related to chemotherapeutic resistance because when WNT5A was knocked down by shRNA, the tumor became drug sensitive; overall, the findings suggest that pathogenesis of castration-resistant prostate cancer (CRPC) is strongly related to the non-classical WNT pathway [96]. Additionally, Hwang et al. carried out CTC transfection using the adenovirus Ad5/35E1aPSESE4 strain, which contains the PSA/PSMA promoter enhancer for specific GFP expression, to identify CTCs. By employing single-cell RNA-seq, this study revealed that MMP9, Cofilin1, and FCER1G are associated with prostate cancer metastasis *in vitro* as well as in animals and patients [97].

### Colorectal cancer

Grillet et al. sequenced CTCs and primary tumors from colorectal cancer patients and found differentially expressed genes between these two groups. Through integrated analysis of previously published breast cancer CTC qPCR data [98], melanoma CTC transcriptome sequencing data [99], and colorectal cancer and prostate cancer microarray data [100], AGR2, CEACAM5, CLDN3, CK18, EpCAM and FGFR3 were found to be commonly differentially expressed between CTCs and primary tumors [101]. In addition, CTCs exhibited higher levels of the stem cell surface markers CD44, CD26 and ALDH1A1 than primary tumors and metastases, indicating that metastatic CTCs might express stem cell biomarkers.

## DISCUSSION AND PERSPECTIVES

After several years of development, single-cell RNA-seq has allowed for breakthroughs in both technologies and applications in oncology research. Despite significant technical noise and low sequencing depth, this powerful transcriptomic tool has greatly influenced and contributed to biomedical research. However, there are limitations and room for improvement in terms of technologies, bioinformatic tools and practical applications in tumor biology.

Currently, single-cell RNA-seq is mainly based on oligo (dT)-mediated reverse transcription of RNAs with poly(A) tails, allowing for the effective removal of ribosomal RNA (rRNA) via negative selection. Nonetheless, this method also excludes regulation-related RNAs without poly(A) tails such as long non-coding RNA and microRNAs. Attempts have been made to overcome the (A) tail restriction, yet the results have not been satisfying. For example, the RCA amplification-based *in situ* fluorescence single-cell sequencing method developed by Lee et al. achieved unselected RNA reverse transcription with random hexamers, but 42.7% of the amplicons were aligned to ribosomal RNA [14]. As another example, SUPeR-Seq sequencing using poly(N) primer enabled accurate quantification of circular RNA. However, due to a theoretical 5′ bias, linear RNAs were also biasedly amplified, and therefore this protocol has not been widely used. Regardless, Ribominus and Ribozero (Thermo and Illumina, respectively) are commercially available due to their ability to simultaneously sequence mRNA and non-coding RNA by removing ribosomal RNA [5, 6, 102]. To our knowledge, there is currently no report of a ribosome removal protocol that is suitable for single-cell RNA application, possibly because of the degradation and loss of RNA during complex steps in library construction and purification. Further improvement of single-cell non-coding RNA-seq will provide a deeper understanding of how gene regulation operates in a given cell type.

A low signal-to-noise ratio is another challenge of single-cell RNA-seq technology. Thus, it is necessary to standardize cell isolation, library preparation, and other automated workflows as much as possible to minimize bias introduced by human error [54, 55]. By measuring the number of detectable transcripts, sensitivity (RNA capture efficiency), and technical noise, the use of standards or standard cell lines can, to some degree, improve the reliability of scRNA-seq. Universal human reference RNA (UHRR) and human brain reference RNA (HBRR) are widely applied as exogenous standards in bulk population RNA-seq (SEQC 2014), and ERCC spike-in is used as an internal references, contributing to technical noise reduction and sensitivity enhancement [103, 104]. Due to the complicated steps involved in cell isolation and library preparation, RNA standards are not ideal mimics of a “real” single cell. Therefore, it is considerably important to establish standard cell lines in future quality control [53, 104].

In the field of cancer cell research, single-cell RNA-seq has the potential to assess the influence of the tumor microenvironment on disease progression, the diversity of tumor antigens and TCR/BCRs, associations between tumor metastasis and CTCs and drug resistance-induced heterogeneity. In the simultaneous presence of immune and non-immune responses, drug-responsive and -sensitive subpopulations can be identified within the tumor tissue, with the combination of tissue decomposition and single-cell RNA-seq approaches [86, 87]. CAFs are thought to be closely associated with the mechanism of drug resistance, and strong expression of complement in CAFs is highly correlated with the number of infiltrating T lymphocyte (TIL) [60]. However, it remains to be determined how tumor cells escape immune monitoring, particularly how CAFs interact with TIL cells. In addition, tumor recognition depends on lymphocyte activation and clonal expansion, which is initiated by TCR and BCR recognition. Sequences of complementary-determining regions on T cell or B cell receptors might correlate with their downstream activities, such as immunotolerance-induced tumor load and the level of chemokine secretion. Nonetheless, the mechanisms by which tumor antigens alter the behaviors of T and B cells and TCR/BCR sequences play function in tumor immunology need to be unresolved [28, 105].

Benefiting from tumor heterogeneity studies, drug development will in time become more accurate. Because the traditional methods of bulk sequencing can mask drug-resistant subclones, targeted and chemotherapy therapeutic effects are limited due to hidden and rare subpopulations with drug-resistance mutations or pathways. Kim et al.'s research highlighted the value of single-cell RNA-seq in tapping every possible drug-sensitive clone and monitoring drug-resistant subgroups at an early stage, as the possibility of recurrence is high with the current first-line chemotherapy and targeted therapy [52].

Liquid biopsy-obtained CTCs should be investigated, as studies by Yu and Miyamoto et al. have shown that ncWNT pathway activation is associated with pancreatic and prostate cancer resistance [91, 96]. CTC transcriptome sequencing can also monitor the EMT phenotype in breast cancer during the course of progression and treatment [96]. In addition, the results of Grillet et al. contribute to diagnosis and prognosis in that they reported a highly metastatic gene biomarker panel shared by CTCs for many cancer types. However, the efficiency and accuracy of CTC capture is still challenging and may affect downstream RNA-seq applications. Ring et al. spiked 10 human breast cancer cell lines into human peripheral blood and used EpCAM target magnetic beads and RNA fluorescence flow cytometry (IE/FACS) to recover the mimic CTCs. This elegant experiment showed that the recovery rate of cancer cells greatly (ranging from 69.5% to 0.004%) depends on the cancer molecular classification, especially with regard to surface markers [106].

With respect to many significant findings in the CTCs, some analyses were based on the comparison between CTCs and the primary tumor tissues. Confounding factors in the impure tumor tissues such as stromal and immune signals may cause biased interpretation. Therefore, comparison between single tumor cells from primary site and CTCs is more reliable to reveal mechanisms like metastasis. Isolation and handling protocols also alter single-cell transcriptome, and should be further addressed. Additionally, as the specificity and sensitivity of the CTC capture rate varies across patients and cancer types, a better bioinformatic tool is required to validate a large number of sequenced CTC samples containing leukocyte, platelet and microvesicle contamination. A shallow sequencing depth is another limitation in this field, which possibly results in a biased evaluation of CTC heterogeneity and dynamics.

Finally, the role of bioinformatics in single-cell transcriptome research cannot be ignored, as the reliability of the bioinformatic method directly determines the accuracy of the experimental results, particularly when results are directly related to drug development or therapy. Accurate definition of tumor cells is also a priority in single-cell RNA-seq data analysis. In a previous study of melanoma tissue dissociation and sequencing, melanoma cells were not detected in 5 of 19 patients, though more unclassified cells were found using the given cell-type characterization algorithm, suggesting that errors or biases in cell classification may exist [60]. The study was the first to utilize a common subset of cells such as T, B and endothelial cells as a negative selection criterion for the tumor; the same method of Petal et al. for tumor definition was applied, in which a 100-genes expression window was extracted to infer the CNV status and thus to define the cell types themselves [61]. Although Hou et al. ultimately demonstrated a correlation between CNV and expression profiles by simultaneous determination of DNA and RNA at the single-cell level, a moderate correlation coefficient is more ambiguous and may result in cell classification bias [86]. Definition of a cancer type by setting a threshold of gene expression level is based on known data, generally from bulk-seq results, for example, PSA, PSMA, and AMACR for prostate cancer. However, further elucidation is required to assess the suitability of this approach for analysis of ab initio cell types and the cancer architecture in a heterogeneous microenvironment [96]. What is more, it is of great challenges to remove confounders such as the technical noises as they might mislead data interpretation. During handling procedures, blood contaminations, apoptosis, necrosis or protocol specific stimulation induced transcriptomic variation should also be studied. Furthermore, it is expected that revolutionary bioinformatic tools will be developed for analyzing single-cell RNA-seq data with exclusive normalization of raw data and measurement of transcriptional kinetics, which is not typically observed in population sequencing data.

Despite the uncertainty of the developing wetlab and drylab protocols, one thing is clear: scRNA-seq is providing new insight into cancer biology. Indeed, single-cell transcriptomic analysis has revolutionized our understanding of gene regulation networks, metastasis and the complexity of intratumoral cell-to-cell heterogeneity, and this technology is expected to eventually benefit patients in a way that has never been available at the bulk level.

## Abbreviations

**Acronym Definition**
CAF: cancer-associated fibroblast
CNV: genomic copy-number variation
CRPC: castration-resistant prostate cancer
CTC: circulating tumor cells
DEP: dielectrophoresis
EMT: epithelial-mesenchymal transition
FACS: fluorescence-activated cell sorting
FFPE: formalin-fixed paraffin-embedded
FISSEQ: fluorescent in situ RNA sequencing
FRISCR-Seq: fixed and recovered intact single-cell RNA-seq
G&T-Seq: genome and transcriptome sequencing
ICA: independent component analysis
IVT: in vitro transcription
LCM: laser-capture microdissection
LNA: locked nucleic acid
MALBAC: multiple annealing and looping-based amplification cycles
MARS-Seq: massively parallel single-cell RNA-seq
MST: minimum spanning tree
NGS: next-generation sequencing
PAGODA: pathway and gene set overdispersion analysis
PDAC: pancreatic ductal adenocarcinoma
PMA: Phi29-mRNA amplification
RCA: rolling circle amplification
RIN: RNA integrity numbers
RRBS: reduced representation bisulfite sequencing
SCDE: single-cell differential expression
scLVM: single-cell latent variable model
scMT-Seq: single-cell methylome and transcriptome sequencing
scRNA-seq: single-cell RNA-Seq
scTrio-seq: single-cell triple-omics sequencing
SMA: semirandom primed PCR-based mRNA transcriptome amplification
SNP: single nucleotide polymorphism
sNuc-Seq: single-nucleus RNA-seq
STRT-Seq: single-cell tagged reverse transcription sequencing
SUPeR-Seq: single-cell universal poly(A)-independent RNA sequencing
SVM: support vector machine
TIL: infiltrating T lymphocyte
TIVA: transcriptome in vivo analysis
TSO: template switching oligonucleotide
UBRR: human brain reference RNA
UHRR: universal human reference RNA
UMI: unique molecule identifiers
WMISH: whole-mount in situ hybridization
